# X‐ray computed tomography for quality inspection of agricultural products: A review

**DOI:** 10.1002/fsn3.1179

**Published:** 2019-08-23

**Authors:** Zhe Du, Yongguang Hu, Noman Ali Buttar, Ashraf Mahmood

**Affiliations:** ^1^ Key Laboratory of Modern Agricultural Equipment and Technology Ministry of Education Jiangsu Province Jiangsu University Zhenjiang China

**Keywords:** agricultural products, computed tomography, nondestructive, quality inspection

## Abstract

The quality of agricultural products relates to the internal structure, which has long been a matter of interest in agricultural scientists. However, inspection methods of the opaque nature of internal information on agricultural products are usually destructive and require sample separation or preparation. X‐ray computed tomography (X‐ray CT) technology is one of the important nondestructive testing (NDT) technologies without sample separation and preparation. In this study, X‐ray CT technology is used to obtain two‐dimensional slice images and three‐dimensional tomographic images of samples. The purpose of the review was to provide an overview of the working principle of X‐ray CT technology, image processing, and analysis. This review aims to focus on the development of the agricultural products (e.g., wheat, maize, rice, apple, beef) and its applications (e.g., internal quality evaluation, microstructure observation, mechanical property measurement, and others) using CT scanner. This paper covers the aspects regarding the advantages and disadvantages of NDT technology, especially the unique advantages and limitations of X‐ray CT technology on the quality inspection of agricultural products. Future prospects of X‐ray CT technology are also put forward to become indispensable to the quality evaluation and product development on agricultural products.

## 1 INTRODUCTION

The agricultural products, including cereals, fruit, and meat, play a significant role in body growth and physiology adjustment of people. And they could provide the calories and nutrition for more than 50% world people (Fischer & Gregoryo, 2010; Herremans et al., 2014; Zhang, Zhu, Xu, & Yue, 2014). The quality is one of the important physical properties in agricultural products, which has long been a matter of interest in agricultural scientists. To detect the high quality of agricultural products well in the market, the quality inspection is very necessary. And the quality inspection of agricultural products includes external inspection and internal inspection. In particular, the information of external inspection, which could be detected, is shape, surface gloss, color, and size. And the physiological changes, insect infestation, relative density, microstructure, moisture loss, and mechanical damage are internal information that could be detected. Due to the opaque nature of internal information on agricultural products, however, the existing inspection methods are usually destructive and require sample separation or preparation (Du & Sun, 2006; Gao & Chen, 2009; Sun & Brosnan, 2003).

Nondestructive testing (NDT) technology has been investigated for a long time, which could improve the production speed, production efficiency, and inspection accuracy with an accompanying reduction of production costs (Chiffre, Carmignato, Kruth, Schmitt, & Weckenmann, 2014; Kruth et al., 2011). The various methods of NDT technology have gradually been the standard detection method by the Association of Analytical Communities, the American Association for Clinical Chemistry, and the International Chamber of Commerce (Wang et al., 2004). Not all NDT methods, nevertheless, indicate the potential ability for the simple operation without sample separation and preparation. And some of these methods are difficult to detect the microstructure, internal crack, bruise, or breakage of samples. Currently, X‐ray CT technology is employed to detect many hidden problems and even the entire information without sample separation and preparation.

X‐ray CT technology, which was made by a combined computer technology with X‐ray technology, was invented in 1972 by British engineer Godfrey N. Hounsfield and American physicist Allan M. Cormack. They were named as “CT's father.” Subsequently, Godfrey N. Hounsfield won a Nobel Prize in 1979, because of the success of CT invention (Hounsfield, 1973). In 1895, X‐ray radiation was first discovered in the Physical Institute of Wurzburg University by Wilhelm Conrad Rontgen. And the first application of X‐ray radiation was employed in the medical field, and the most compelling application for CT was the medical diagnosis. Particularly, the special need of medicine had driven the progress of CT theoretical research and equipment (Bertram, Kohler, Bocker, Ofstad, & Andersen, 2006; Lent, Vanlerberghe, Oostveldt, Thas, & Meeren, 2008; Li, Long, Liu, & Zhao, 2018). With the development of CT technology and various professional disciplines in recent years, numerous applications and some reviews had been published to prove the importance of X‐ray CT technology in the field of instance, industry, engineering, safety testing, and agricultural science (Anonymous, 2010; Ding, 2005; Shao, Cao, & Wang, 2006; Sun, Liang, Hu, & Xu, 2008; Sun & Ye, 2006; Zhao et al., 1996).

Interest in CT technology utilized in the agricultural field led to rapid interest in the visualization of inner dynamic conditions among agricultural researchers. The development of X‐ray CT technology is transformed from external quality inspection to internal quality inspection. This technology is encouraged to inspect and visualize the internal information on two‐dimensional (2D) structure and three‐dimensional (3D) structure of agricultural products (Fox & Manley, 2009; Girvin & Gupta, 2006; Guelpa, Plessis, & Manley, 2016). The pore topology, tiller numbers, internal corruption, grain hardness, moisture, temperature, volumes and densities, and mechanical damage could be achieved with CT scanner (Arendse, Fawole, Magwaza, & Opara, 2016; Chen, Xu, Yin, & Tang, 2017; Dhondt, Vanhaeren, Loo, Cnudde, & Inze, 2010; Longuetaud, Leban, Mothe, Kerrien, & Berger, 2004; Stuppy, Maisano, Colbert, Rudall, & Rowe, 2003; Yang et al., 2011; Yu & Qi, 2007). X‐ray CT technology is becoming a routine tool for the nondestructive 3D characterization and the reconstruction of tomographic imaging. Takhar et al. analyzed the transport routes and transport mechanisms of moisture on maize grains using micro‐CT scanner to solve the stress equation for predicting the drying parameters at the intermittent drying conditions. And the experimental average moisture content could be expected by the model with reasonable accuracy of *R* ^2^ =* *.88–.99 and CV =* *3.5%–9.5% (Takhar, Maier, Campanella, & Chen, 2011). X‐ray CT technology was utilized to observe the formation of stress cracks in maize grain that could be caused by high temperature and excess moisture (Carvalho, Aelst, Eck, & Hoekstra, 1999). The quality information of agricultural products could be inspected nondestructively by X‐ray CT technology.

In this paper, we are focused on the X‐ray CT technology for the quantitative and qualitative analysis of internal structure and components with cereals (wheat, rice, and maize), fruit (apple and pear), and meat (beef and lamb). This review demonstrates that the recent progress of X‐ray CT as a nondestructive technology for 2D and 3D investigations related to agricultural products. The theory and systems of X‐ray CT technology are briefly reviewed. The core content discusses the previous research and applications of agricultural products, unique advantages, and limitations of NDT technology on the quality inspection of agricultural products. In addition, the future trends of X‐ray CT technology are discussed for the quality inspection and product development on agricultural products.

## 2 WORKING PRINCIPLE OF X‐RAY CT TECHNOLOGY

### 2.1 Basic principle

The first prototype of the CT scanner was installed to examine the first patient in the Atkinson Morley Hospital. A typical CT equipment consists of three parts: scanning part, computer system, and image system. The scanning part is made up of the X‐ray source, detector, and gantry. And the data, which are collected by the scanning part, are stored on the computer system. Besides, image system displays the computer‐processed and reconstructed images on a screen (Neethirajan, Jayas, & Ndg, 2007; Wang & Wang, 2001).

The core principle of X‐ray CT technology, which was introduced in 1917 by Johann Radon, was Radon transform. Based on the attenuation of a transmitted X‐ray beam, X‐ray CT technology has different mass attenuation characteristics with a consequence of the different materials comprising samples (Kays, Barton, & Windham, 2000a; Kotwaliwale, Weckler, & Brusewitz, 2006; Kotwaliwale et al., 2014; Tretiak & Metz, 1980). According to the decay characteristics of X‐ray on the material, the working principle of X‐ray CT technology is to obtain the radiation attenuation information when X‐ray passes through the sample at different directions. The decay characteristics of different materials represent different selected X‐ray energies. The X‐ray energy of medical radiation is generally 140 keV, while that of industrial CT is selected as 50 keV–16 MeV. In the inspection of agricultural products, the selected energy is usually as variable as variety, and in some cases more so.

**Figure 1:**
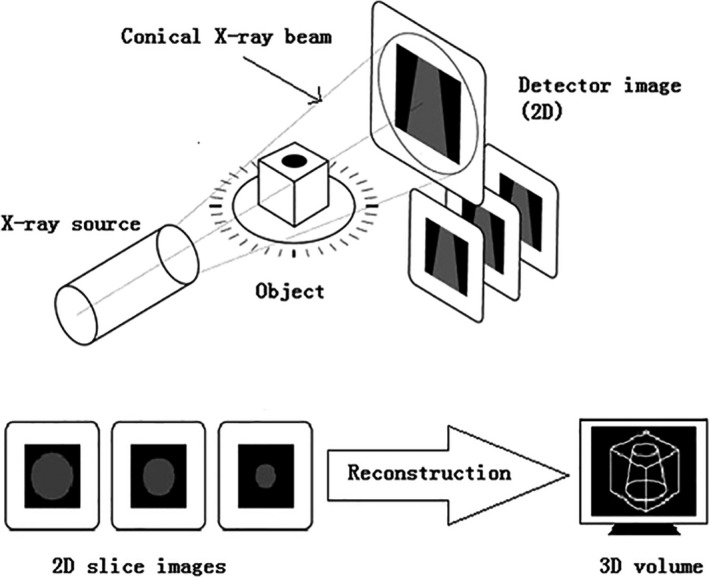
Working principle of X‐ray CT technology. Some radial projections are captured at different angles on a sample by the X‐ray to obtain 2D slice images. Besides, a 3D image could be produced by the restructure of scanned numerous slice images of the sample

The traditional CT is an imaging procedure where an X‐ray tube rotates around a sample or samples and then the attenuation is recorded on a detector. The focus of X‐ray tube limits the spatial resolution, while actual resolution depends on the product size and its magnification times. Some radial projections are captured at different angles on a sample by the X‐ray, usually completing a 180‐degree rotation. Generally, a charge‐coupled device camera is applied as an enlarged radiography. Afterward, it is reasonable to obtain 2D slice images from different angle projections and then to superimpose the sequence of 2D slice images for obtaining 3D volume (Cosmi & Bernasconi, 2013; Wang, 2004). The formation processes of CT images include image scan stage, reconstruction stage, and display stage. For CT images, voxel and pixel are the basic structural unit of an image and the basic unit of the image on a screen, respectively. The image clarity depends on the voxel and pixel. Besides, a 3D image could be produced by the restructure of scanned numerous slice images of the sample (Mooney, Pridmore, Helliwell, & Bennett, 2012; Stock, 2008; Zhuang, 1992), as shown in Figure 1.

X‐rays are both invisible light and electromagnetic waves. X‐ray is produced by a speeding electron impact on a sample, where the energy of X‐ray photon is equal to the total kinetic energy of the electron. X‐ray photon energy *E* could be defined as follows:

(1) $$E=hv=\frac{\mathrm{hc}}{\lambda },$$

where *h* is Planck constant, Js;

*c* is light velocity, m/s; and

λ is X‐ray wavelength.

Usually, the unit of X‐ray photon energy *E* is eV, and 1 eV = 1.62 × 10^–34^ J. *h* and *c* are approximately equal to 6.63 × 10^–34^ Js and 3 × 108 m/s, respectively. Most X‐rays have a wavelength ranging from 0.1 mm to 0.01 nm corresponding to energies in the range of 10 eV to 450 keV. Photons in a soft X‐ray beam, when these pass through a sample, are being absorbed, transmitted, or scattered. And in general, the higher the material density and atomic number of constituents are, the greater the X‐ray absorption is. As a result, the attenuation intensity is indicated by the Beer–Lambert law (Curry, Dowdey, & Murry, 1990; Wu, Yang, & Zhou, 2008; Ying & Han, 2005).

(2) $$I=I_{0}e^{-\mu_{n}l},$$

where *I* is the intensity of X‐ray exiting through a sample in eV;

*I*_0_ is the intensity of X‐ray in eV;

*μ*_*n*_ is the linear attenuation coefficient of a sample on the wavelength in eV/mm; and

*l* is the path length through a sample in mm.

In CT system, the material difference of physical density could be visualized by the changes of image intensity. And X‐ray attenuation capabilities seem to reflect the difference. Moreover, these capabilities on voxel could be represented as CT number in a 3D image. The CT number is the attenuation coefficient which is calculated using water, and its unit is Hounsfield (Hu) (Cnudde et al., 2006; Donis‐Gonzalez, Guyer, Pease, & Barthel, 2014; Taina, Heck, & Elliot, 2008). The calculation formula of CT number is as follows:

(3) $$\text{CT number}=\frac{\mu -\mu_{w}}{\mu_{w}}\times 1000,$$

where *μ* is the linear attenuation coefficient of a sample, and

*μ*_w_ is the linear attenuation coefficient of water (approximately 0.195).

The measurement range of CT number is from –1,000 Hu to + 4,000 Hu. Besides, CT number for air is –1,000 Hu and the number for water is 0 Hu (Ogawa, 1998).

### 2.2 Image processing and analysis

The internal information of agricultural products includes the microstructural information (cell size, cell wall size, pores number, and porosity), and physical, chemical, and mechanical properties (Jha, Kingsly, & Chopra, 2006; Schoeman, Williams, Plessis, & Manley, 2016). The image processing is required to extract the suitable information from images and to obtain CT number accurately. Then, the accurate visualization and inner component identification could be carried out using analysis of CT images. By combining with the image processing technology, image analysis could provide the information of the microstructural variations on agricultural products (Leonard, Blacher, Nimmol, & Devahastin, 2008).

**Figure 2:**
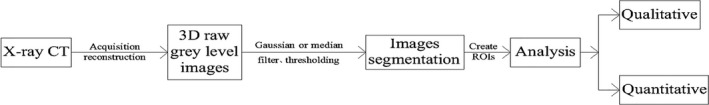
Image processing and analysis procedure. 2D slice images are generated to reconstruct the 3D model. Gaussian or median filter is used to reduce noise with 3D raw gray‐level images. The threshold segmentation is used to segment the image based on the gray value histogram of different regions. The final step is the qualitative and quantitative analysis on CT data of ROIs

In order to distinguish phase materials (air and samples), the image segmentation is used to separate the voxel group of region of interests (ROIs). The image processing technology is usually performed with threshold segmentation. The textural features of CT images could be extracted using histogram groups, histogram, and shape moments to identify the ROIs on the sample (Karunakaran, Jayas, & White, 2003a, 2003b). And some small quantities of pixels could be removed by a cleaning step (Baker et al., 2012). The random noise, detector defect, and artifact defect appear in 2D slice images after image segmentation. For reducing the noise and correcting the defects, the median filter, Gaussian filter, and noise reduction technology are used (Frisullo, Laverse, Marino, & Nobile, 2009; Shahin, Tollner, & Prussia, 1999). According to the matrix arrangement, CT images are composed of different grayscale pixels from black to white. Hence, the area of high‐attenuation regions (high Hu) is brighter and the area of low attenuation (negative Hu) is darker. Because of the varying distribution in density regions of a sample, the internal structure of agricultural products could be visualized and analyzed through 3D tomographic images of volume rendering. The image analysis is used to obtain the quantitative and qualitative information on the sample. Currently, some software, such as ImageJ, Avizo, Amira, and VGStudio Max, are usually used to analyze the quantitative, qualitative, and statistical data for sample structure (Mao, Kumi, Li, & Han, 2016). Image processing and analysis procedure was illustrated by Schoeman et al. (2016), while a maize grain would be imaged and analyzed. Firstly, 2D slice images are generated to reconstruct the 3D model. Then, 3D raw gray‐level images reduce noise with Gaussian or median filter. Meanwhile, the threshold segmentation is used to segment the image based on the gray value histogram of different regions. The final step is the qualitative and quantitative analysis on CT data of ROIs. A typical image processing and analysis procedure is schematically depicted in Figure 2.

## 3 X‐RAY CT APPLICATION ON AGRICULTURAL PRODUCTS

In recent years, X‐ray CT technology has become a useful tool to assess the quality of agricultural products, enabling a better understanding of the composition, physicochemical characteristic, and internal structure on samples. More specifically, an extensive range of agricultural products such as cereal (wheat, corn, and rice), fruit (apple and pear), and meat (beef and lamb) could be analyzed by X‐ray CT technology with the high‐resolution 2D and 3D visualization. And the applications include internal quality evaluation, microstructure observation, mechanical property measurement, and others (Schoeman et al., 2016). The 2D structure of agricultural products could be observed with 2D slice images using CT scanner. Besides, the 3D model, which merges numerous 2D slice images, could be established (Trinh, Lowe, Campbell, Withers, & Martin, 2013). An overview of CT applications related to various agricultural products is shown in Table 1.

**Table 1:** Previous study on the use of CT in agricultural products. An overview of quality inspection of various agricultural products using X‐ray CT is shown. The quality inspection includes internal quality evaluation, microstructure observation, mechanical property measurement, and others. The agricultural products are wheat, corn, rice, apple, pear, beef, and others

Food type	Specific requirement	Focus of research	Area(s) for further research	References
Internal quality evaluation				
Mango	150 keV, 3 mA	CT number, moisture, pH, and soluble solids	Internal quality evaluation	Barcelon et al. (1999)
Apple	–	Moisture and CT number	Study the drying process	Zhang, Kong, Zhu, and Zhang (2013)
Pork	130 keV, 6.2 mm	Salt concentrations	Optimal salt distribution and minimal production time	Vestergaard et al. (2004)
Apple	–	CT number and PH	Acidity prediction	Zhang, Liu, and Wang (2009)
Apple	110 keV, 30 mA	Acidity, moisture, and CT number	Predict and analyze apple quality	Huang et al. (2013)
Apple	120 kV, 150 mA, 3 mm	Bitter pit	Eliminate pitted fruits prior to packaging and transportation	Jarolmasjed et al. (2016)
Wheat	15 kV, 65 μA	Infected grains	Identification of uninfected and infected wheat grains	Karunakaran et al. (2003a)
Wheat	15 kV, 65 μA	Identify infected grains	Uninfected and infected grains using extracted features	Karunakaran et al. (2003b)
Wheat	140 kV, 96 mA, 3.42 mm	Internal infestation of insect‐damaged	Recognize and quantify infected grains	Toews, Pearson, and Campbell (2006)
Wheat	13.5 kV, 185 mA, 26 kV, 11 mA, 60 mm	Gray‐level distribution of sprouted grains	Detection of sprouted and healthy wheat grains	Neethirajan et al. (2007)
Rice	–	Moisture and temperature	Modeling of mass transfer and initiation of hygroscopically induced cracks	Perez, Tanaka, and Uchino (2012)
Wheat	26 kV, 300 mA, 25 μm	Mass loss in grains	Mass loss determination of wheat grains infected	Nawrocka, Stepien, Grundas, and Nawrot (2012)
Wheat	–	Determining the quality of damaged kernel	Detection of the granary weevil of damaged wheat grains	Bonieckia et al. (2014)
Apple	50 kV, 200 mA	Microstructure and ice crystal distribution	Characterize 3D microstructure of frozen apple	Vicent et al. (2017)
Apple	80 kV, 439 mA	Bruise volumes	Assessment of bruise volume	Diels et al. (2017)
Microstructure observation				
Pear	53 kV, 0.21 mA	Cavity and core area per slice	Spatial distribution of core breakdown disorder symptoms	Lammertyn et al. (2003)
Rice, fat, etc.	–	Microstructure, size, shape, and networking	3D image of large samples, microstructure of food	Dalen et al. (2003)
Wheat, peas, etc.	420 kV, 1.8 mA, 120 mm	Air‐path area and air‐path lengths	Explain the airflow resistance differences	Neethirajan, Karunakaran, Jayas, and White (2006)
Rice	50 kV, 100 mA, 9.1 mm	Micropores, cell walls, and macropores	Structural and hydration properties of heat‐treated rice	Witek et al. (2010)
Rice	28 keV, 9 μm	Pores number, porosity, and specific surface area	Investigate 3D microstructure of soil aggregates for rice yield	Zhou et al. (2011)
Rice	46 kV, 75 mA, 3.91 mm	Microstructure, endosperm structure, and air space	3D characterization of rice grain structure	Zhu et al. (2012)
Wheat	17.6 keV, 5 μm	Porosity, connectivity index, bubble size, and cell walls thickness distributions	Describe phenomena involved in the growth of bubbles in dough during fermentation	Turbin‐Orger et al. (2015) (Turbin‐Orger et al., 2015)
Cereal	50 kV, 800 mA, 6.46 mm	Microstructure of agglomerated cereal	Understand 3D internal morphology of food agglomerates	Hafsa et al. (2014)
Cereal	17.6 keV, 50 kV, 6.5, 7.5, 16.2 mA, 25.8 mm	Cells and cell walls	Determine cellular structure of cereal	Chevallier et al. (2014)
Pome	700 nm	Cortex tissue, cell wall, pore network, and cells	3D microstructure modeling of fruit tissue	Mebatsion et al. (2009)
Fruit	–	Parenchyma tissue	A new model for effective oxygen diffusivity of parenchyma tissue	Herremans et al. (2015)
Wheat	40 kV, 250 μA	Porosity, anisotropy, and absolute permeability	Determine internal structure of wheat to predict moisture transport and viscoelastic stresses	Suresh and Neethirajan (2015)
Rice	40 kV, 100 mA, 6 μm	Internal structure, texture properties, starch, and proteins	Impact of extrusion parameters on the properties of rice products	Chanvrier et al. (2015)
Wheat	60 kV, 240 μA, 12 μm	Volume, porosity, expansion ratio, and relative density	Roast on microstructure of wheat grains	Schoeman et al. (2016)
Mechanical property measurement				
Apple	–	CT number	Study on CT number of damaged apple	Xu, Yu, and Wang (2006)
Wheat	420 kV, 1.8 mA, 120 mm	Hardness	Classifying vitreous or nonvitreous grains	Neethirajan et al. (2006)
Corn	–	Texture, mechanical properties, and structure	Microstructure of flakes and morphology of their constitutive materials	Chaunier, Valle, and Lourdin (2007)
Pears, apple, etc.	70 mA	CT number	Detection of mechanically damaged fruits	Wang, Xi, and Wang (2008)
Wheat	–	Tiller number	Wheat tiller inspection	Jiang et al. (2012)
Maize	60 kV, 13.4 μm	Hardness and density	Optimum quality and yield during the milling process	Guelpa et al. (2015)
Corn	–	Internal stress	Internal stress value, distribution, and cause of stress	Liu, Kong, Zhang, and Zhang (2015)
Food	45 kVp, 177 μA, 35.6 mm	Density	Density of calculation from X‐ray linear attenuation coefficients	Kelkar et al. (2011)
Asparagus	120 kV, 120 mA	Tough‐fibrous tissue	Classification of tough‐fibrous asparagus	Donis‐Gonzalez et al. (2016))
Wheat	61 μm	Stem diameter, thickness, tiller number, and angle	Morphological trait extraction of wheat tillers	Wu et al. (2017)
Wheat	–	Geometric features	Wheat grain classification	Charytanowicz, Kulczycki, Kowalski, Lukasik, and Czabak‐Garbacz (2018)
Other applications				
Salami	82 kVp, 125 μA	Structure thickness, structure–volume ratio, and percentage volume	Study processed meat microstructure	Frisullo et al. (2009)
Beef	60 kVp, 167 μA	Intramuscular fat	Assessment of intramuscular fat level and distribution in beef muscles	Frisullo, Marino, Laverse, Albenzio, and Nolile (2010)
Lamb	–	Intramuscular fat content	Prediction of fat and conformation grade	Lambe et al. (2017)

Note

Specific requirement includes voltage, current, and detector resolution requirement.

### 3.1 Internal quality evaluation

At present, X‐ray CT technology has been applied in the internal quality evaluation of agricultural products such as maturity, sugar, acidity, oil content, and tissue breakdown. The CT number of slice images is an important index to evaluate the internal quality. CT number followed a positive linear relationship with moisture content, acidity, and density, while soluble solids and PH had a negative linear relationship with CT number (Barcelon, Tojo, & Watanabe, 1999; Donis‐Gonzalez et al., 2014). Huang et al. established the models of CT number with sugar, moisture, and titratable acidity, and *R* ^2^ value of models was .8464, .9075, and .8233, respectively, within 8% of the prediction error. The moisture content increased with CT number, while the values of sugar and titratable acidity decreased. At this moment, the scanning parameters of CT scanner were X‐ray source voltage of 110 kV and current of 30 mA (Huang, Sun, & Zhang, 2013). A similar conclusion was achieved with the study on peaches, asparagus, mango, and others. They have similar source voltage and different current (Donis‐Gonzalez, Guyer, & Pease, 2016; Jha et al., 2006).

**Figure 3:**
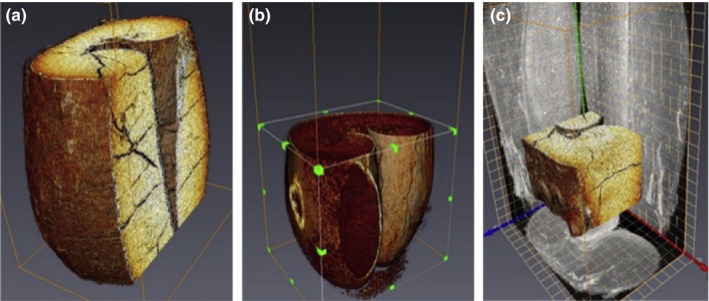
3D visualizations of wheat grain (Suresh & Neethirajan, 2015). (a) Vertical cross section of insect‐infected wheat grain; (b) horizontal cross section of sprout‐damaged wheat grain; (c) region of interest in the insect‐infected wheat grain alongside its ortho‐projection

For fresh agricultural products, the visual sense and textural difference have an impact on the consumption. In 1970, Diener, Mitchell, and Rhoten (1970) found a new method to sort the bruise and health of apples using X‐ray CT technology. A similar study was obtained when Diels et al. (2017) detected, quantified, and assessed the bruise volumes automatically to classify the bruise and health of apples. The results showed that CT number decreased with the increase in the destruction degree on apples. In addition, X‐ray systems have an obvious effect on the detection of insect infestation. The internal features of wheat grains with the insect infestation or sprouting could be investigated with X‐ray CT technology for 3D visualization and quantification analysis (Figure 3) (Suresh & Neethirajan, 2015). Karunakaran et al. (2003a) used the X‐ray CT technology and backpropagation neural network (BPNN) with the histogram, features, textural features, and shape moments to classify the uninfected and infected wheat grains precisely. A similar method was used when Neethirajan, Jayas, and Karunakaran (2007) detected and classified the difference of the gray‐level histogram in density between healthy and sprouted grains with CT images. In order to identify the different CT number between the healthy and defective cucumber as well as healthy and rotten chestnuts, the grayscale variations in images could be observed by CT technology and image processing technology according to the intensity differences in different regions on 2D slice images (Guelpa et al., 2016).

The bitter pit is an important physiological disorder with roundish brown lesions, resulting in serious economic losses for apples. To identify the internal symptoms of apples, X‐ray CT technology and image processing algorithms were used by Si and Sankaran (2016) to acquire the qualitative and quantitative data from the sample with bitter pit. In the same way, Honeycrisp apples with bitter pit before and after harvesting could be identified with CT slice images. And the classification accuracy of bitter pit and healthy fruit ranged between 70% and 96% (Jarolmasjed, Espinoza, Sankaran, & Khot, 2016). Moreover, X‐ray CT technology is also applied for the quantification of salt concentrations on meat (Vestergaard, Risum, & Adler‐Nissen, 2004).

### 3.2 Microstructure observation

**Figure 4:**
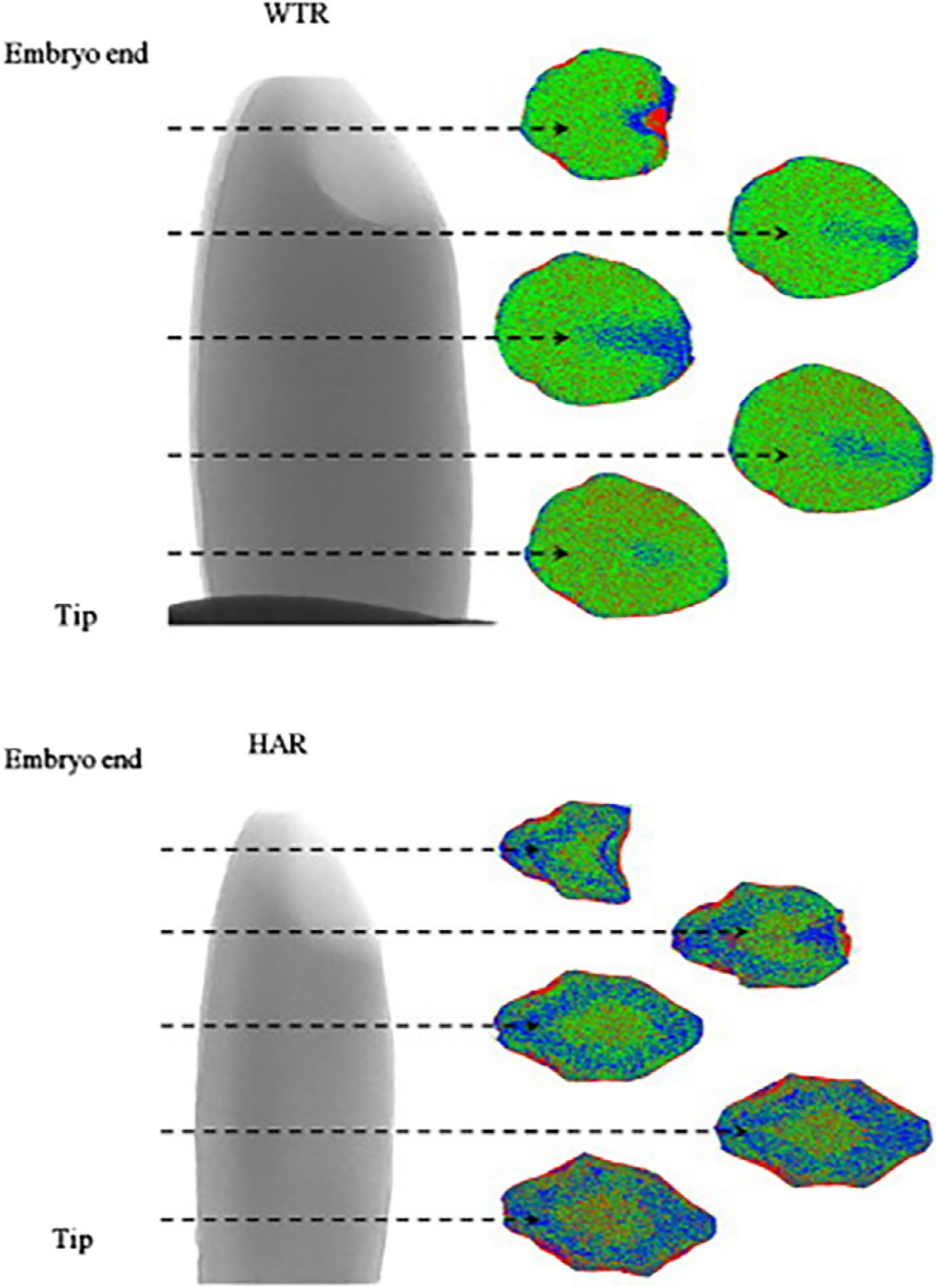
Spatial distribution of density within kernels by X‐ray CT. WTR is wild‐type rice; HAR is high‐amylose rice (Zhu et al., 2012). The instruments are SEM with an accelerating voltage of 30 kV and a high‐resolution X‐ray CT system with operating voltage of 46 kV and current of 75 μA. The amount of void was the least near the tip part of the WTR grain and varied throughout grain. Within the HAR grain, the part near the tip also had the least amount of void, but no difference was found in other parts of the grain

The microstructure of agricultural products could determine their physical texture and sensory properties, which could be observed by CT scanner. The microstructure, particularly the porosity, connectivity index, and cell wall thickness distribution, is needed to study correctly for understanding the mechanical properties and organoleptic properties of agricultural products (Aguilera, 2005; Dalen, Han, Aalst, & Hendriks, 2003). There are some papers that have been published about nondestructive inspection of agricultural products’ microstructure, as shown in Table 1. Zhu et al. acquired the spatial distribution of density on rice grains by an environmental scanning electron microscope (SEM) at an accelerating voltage of 30 kV and a high‐resolution X‐ray CT system (Figure 4). In X‐ray CT system, the X‐ray tube had a spot size of 5 mm with operating voltage of 46 kV and current of 75 μA. The high‐resolution 2D microstructure and 3D structural characterization of rice grain could be provided by SEM and CT images, respectively (Zhu et al., 2012). Spatial distribution of core breakdown disorder symptoms in pears could be analyzed by a useful technology which included the magnetic resonance imaging (MRI) and X‐ray CT technology (Lammertyn et al., 2003).

Due to the water demand of starch mass and the porous microstructure of rice grains, Mohoric et al. (2009) found mesostructure of grains had a positive correlation with its microstructure. Roast has a direct impact on the microstructure of agricultural products. Schoeman et al. (2016) investigated the roasted wheat grains using CT images to find that the volume, porosity, and expansion ratio gradually increased with the decreased of relative density. Furthermore, Mebatsion et al. (2009) used synchrotron X‐ray CT scanner and transmission electron microscopy to establish 3D models with three important components of fruit cortex tissue, cell wall, pore network, and cells. A more study by Schulze, Peth, Hubbermann, and Schwarz (2012) found that the differences of pore size distribution, porosity, stiffness, and cell morphology in the tissue of apples had a strong influence on enrichment process. X‐ray CT technology makes an inspection on the microlevel, which is beneficial to the agricultural products because the transport properties of tissues, cellular structure, dimension, and distribution of pores could lead to the improvement of physical and sensory properties (Chevallier, Reguerre, Bail, & Valle, 2014; Vicent, Verboven, Ndoye, Alvarez, & Nicolai, 2017).

### 3.3 Mechanical property measurement

The tiller number is one of the most essential agronomic traits and mechanical properties, which determines the wheat and rice yield (Li et al., 2003; Xing & Zhang, 2010). The 3D structure of tillers could be reconstructed by the algorithms of filtered back‐projection and graphics processing unit with CT images (Wu, Yang, Niu, & Huang, 2017). Yang et al. developed a high‐throughput equipment based on X‐ray CT technology to measure the tiller numbers automatically. For saving the time‐consuming reconstruction, the optimized method of adaptive minimum enclosing rectangle was employed in this equipment to measure the tiller numbers with high accuracy according to the requirements of real‐time imaging (Arendse et al., 2016; Jiang et al., 2012). Furthermore, the nondestructive inspection of morphological characteristic parameters, such as tiller number, tiller angle, tiller stem thickness, and tiller wall thickness, could be achieved by X‐ray CT technology.

**Figure 5:**
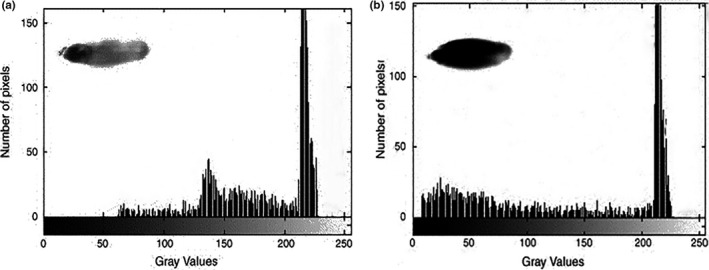
Histogram of durum wheat grain transmitted light images. (a) Vitreous; (b) nonvitreous. (0 represents black and 255 represents white in the *x*‐axis) (Neethirajan et al., 2006). The images were acquired by X‐ray CT at 17 kV potential, 65 μA current, and a resolution of 60 pixels/mm. Nonvitreous kernels have more optically dense regions than the translucent vitreous kernels

**Figure 6:**
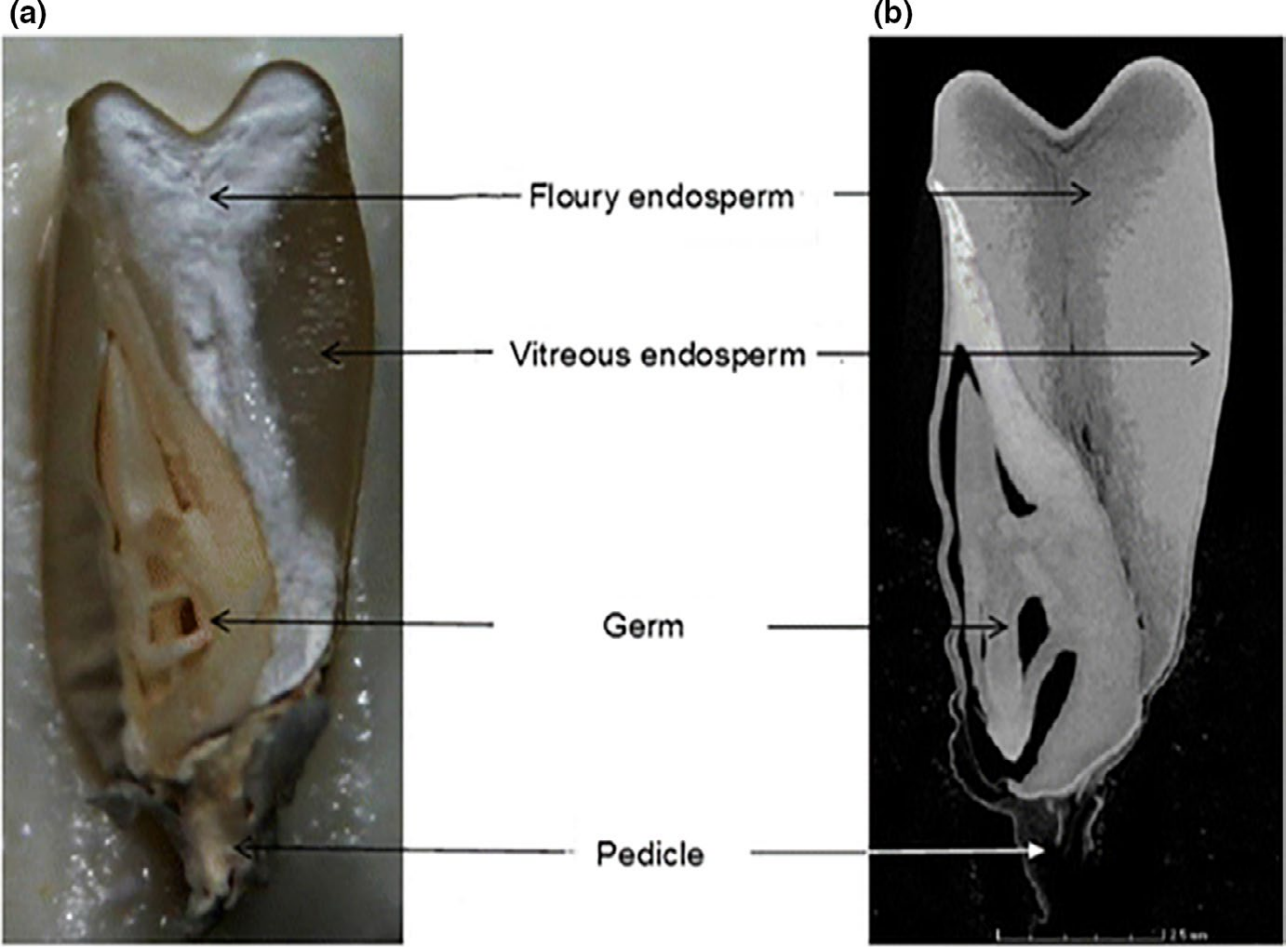
Comparison of a longitudinal digital image and CT image slice. (a) A longitudinal digital image; (b) 2D CT image slice (Guelpa et al., 2015). The voxel size, voltage, and scan time are 13.4 μm, 60 kV, and 30 min, respectively. The same maize grain is depicting the internal structure of the maize grain, that is, flour and vitreous endosperm, germ, and pedicle. In CT image, the brighter gray region represents the denser vitreous endosperm and the darker region the less dense floury endosperm. The vitreous endosperm thus appeared translucent (Figure a) due to no light being reflected

In addition, the physical and mechanical properties of agricultural products also include color, size, texture, shape, density, and hardness (Witek et al., 2010). However, some features have different types of values based on the different measuring way. And the inspection on physical and mechanical properties of agricultural products becomes real challenges without a standard method (Dhondt et al., 2010). Currently, X‐ray CT technology has the great potential to estimate and measure the density and porosity in comparison with traditional technologies (Kelkar, Stella, Boushey, & Okos, 2011). Neethirajan et al. found that the wheat grains with different strains could be represented with the differences of starch granule shapes and pore shapes. The images were acquired by X‐ray CT at 17 kV potential, 65 μA current, and a resolution of 60 pixels/mm. Figure 5 shows that the vitreous and nonvitreous of durum grains could be identified and classified by X‐ray CT technology and transmitted light system (Neethirajan, Karunakaran, Symons, & Jayas, 2006). In order to determine the pore interconnectivity, strut size, porosity, and pore size as well as overall 3D micro‐architecture, Darling and Sun reconstructed 3D models with CT‐scanned serial images. The micro‐CT scanner had a focal spot size of several microns with operating at 100 kV and 19.1‐μm resolution (Darling & Sun, 2004). The potential function of X‐ray CT technology was studied by Gustin et al. (2013) to determine the volume and density of maize grains. Figure 6 displays the internal structure of maize grains by CT scanner at voltage of 60 kV and voxel size of 13.4 μm. Guelpa, Plessis, Kidd, and Manley (2015) calculated the density, percentage porosity, and percentage cavity of maize grains to estimate the grain hardness from CT images. With the development of CT technology, the cost‐effective and less time‐consuming methodology was introduced to measure the entire volumes and densities of maize grains (Stuppy et al., 2003). Additionally, the internal stress, interior heat, and mass transfer are the cause of the variations in physical and mechanical properties on agricultural products, which could be viewed on CT images.

### 3.4 Others

From the previous research, X‐ray CT technology is rapidly becoming a very useful technology for the measurement and analysis of internal structure on agricultural products, such as fat content, fat distribution, fat microstructure, extruded starches, lean meat content, lean weight, and meat yield (Hollo, Szucs, Tozser, Hollo, & Repa, 2007; Jay, Van, & Hopkins, 2014). To increase the meat eating quality, Lambe et al. (2017) used CT slice images of packaged lamb cuts to predict the intramuscular fat content. Based on the best CT prediction equation, the prediction level of samples was higher on average than this prediction by the taste panel. Furthermore, X‐ray CT technology could be applied to visualize the ice crystal structure formation during the freezing of meat, chicken, fish, and carrot (Mousavi, Miri, Cox, & Fryer, 2007). A similar method was utilized when Haseth, Kongsro, Kohiler, Sorheim, and Egelandsdal (2008) measured the quantity of sodium chloride in ground pork and dry‐cured hams with CT images at different voltages (80, 110, and 130 kV). Meanwhile, some experts focused on the soil aggregate structure of rice to increase the rice yield. Li, Zhou, Chen, Peng, and Yu (2014) found that the combined application of organic manure and chemical fertilizer could build up soil fertility to maintain the soil in good aeration for improving the yield. In practice, some CT applications have proved to predict intramuscular fat content from meat cuts of pork, beef, and lamb, and even vacuum‐packaged meat (Furnols, Brun, Tous, & Gispert, 2013; Prieto et al., 2010). In addition, CT's security inspector is an important application that you can use to effectively identify organic, inorganic, and mixtures in baggage for security check in civil aviation airport, customs, and large‐scale exhibition.

X‐ray CT technology enables examination at the microstructural level, which is useful to the agricultural products, as the accurate calculation of the moisture content, density texture, hardness, and density could lead to improve the ability of internal quality evaluation, microstructure observation, and mechanical property measurement. And they could be inspected with the quality feature by this technology extending to other products besides the inspection of quality feature on some agricultural products in this paper. The CT scanner is suitable to be popularized and applied in the quality inspection of products. X‐ray CT technology, as a novel technology, has much literature focus on characterizing the agricultural products from scientific point of view in the laboratory and not in a commercial environment. For larger cultivated area and plant diversity of agricultural products, the portability and operability of X‐ray CT equipment should be taken into account to enhance the quality inspection efficiency and accuracy. Thus, the application of this technology in agricultural production line has room for future investigation and development.

## 4 COMPARISON OF X‐RAY CT AND OTHER NDT TECHNOLOGIES

In recent years, NDT technology is an emerging high‐tech inspection technology for quality inspection of agricultural products. The properties of agricultural products, such as physical, electrical, thermal, electromagnetic, mechanical, optical, and sonic, are utilized with NDT technology to inspect the maturity degree, sugar content, sugar–acid ratio, moisture detection, fat content determination, contaminants determination, product classification, and physical inspection (Li, Rao, & Ying, 2011). Currently, among NDT technology used in research we usually do as follows: near‐infrared spectroscopy (NIRS), terahertz time‐domain spectroscopy (THz‐TDS), nuclear magnetic resonance (NMR), and X‐ray computed tomography (X‐ray CT) technology. Different technologies have their unique advantages in separate fields. This paper covers the aspects regarding the comparison of X‐ray CT technology and other NDT technologies.

1. By combining with chemometrics method, NIRS technology could detect the quality parameters (e.g., internal quality, microstructure, mechanical properties) to establish the model with some variables in agricultural products (Kays, Barton, & Windham, 2000b; Manley, Van, & Osborne, 2002; Osborne, 2000). NIRS methods have great development over the decades; however, this technology still has some problems. For instance, light conditions, sample inspection location, and loading conditions are under a strong impact on the NIRS information. Construction of NIRS model requires substantial development effort; meanwhile, the model accuracy will be affected by the modeling technologies of user (Wang et al., 2004).
2. Because of the transient, broadband, and low‐energy properties, Thz‐TDS technology has received a great attention to agricultural scientist in the nondestructive test (Qin, Ying, & Xie, 2013; Saha, Grant, Khalid, Hong, & Cumming, 2012). Generally, agricultural products have the water‐bearing characteristics, while THz waves have a strong sensitivity to moisture. The necessary programs before testing are dehydration and drying. Besides, a mass of repeated test is required to realize a high precision of modeling (Sun et al., 2008).
3. NMR technology may often yield different internal information of agricultural products compared with other NDT technologies (Shao et al., 2006; Zhao et al., 1996). However, there is less literature about this technology that has focused on characterizing the agricultural products. Even though NMR technology has proven to be a highly versatile imaging technique, this technology has some inherent difficulties, like complex data processing and low signal‐to‐noise ratio. Moreover, compared with CT scans, MRI scans typically take much longer to treat.
4. Compare with other NDT technologies, X‐ray CT technology has unique advantages for the above problems. The advantages of X‐ray CT technology include the following (Donis‐Gonzalez et al., 2014; Kumi, Mao, Hu, & Ullah, 2015; Wang, 2004): (a) This technology has a high density and space distinguishable abilities to obtain the high‐quality images clearly and quickly. (b) This technology could measure the X‐ray absorption attenuation of each sample tissue without sample preparation and separation.

X‐ray CT technology has a few limitations in quality inspection of agricultural products. Because the image scanning and 3D image reconstruction need a long computation time and low speed, the CT applications for real‐time imaging are limited (Herman, 1995). The cost of CT equipment and the corresponding system is the main question, especially for a wide variety of agricultural products with generally low prices. In addition, CT equipment only could be employed in the cross‐sectional scan, because of the limitations on the hardware structure (Cnudde & Boone, 2013).

## 5 FUTURE PROSPECTS

Based on the review of agricultural products with X‐ray CT technology, the following need to develop for the future.

The scanning time of X‐ray CT equipment remains a concern. A single maize kernel needed 30‐min scanning time at the resolution of 13.4 μm, and while 2–3 hr were needed to obtain the resolution of 6 μm (Guelpa et al., 2015). The moisture content, mechanical damage, internal stress, and even microstructure of agricultural products are changed with scanning time, which leads to collect inaccurate data by CT equipment. Besides, the image analysis is also a time‐consuming work in the use of CT technology (Schoeman et al., 2016). Development of models and algorithms further reduce the consumption time of image acquisition and analysis on the complex internal structure of agricultural products.

Different X‐ray CT parameters such as the tube voltage, current, and exposure time are chosen at random, and this ultimately affects the image quality (Cnudde & Boone, 2013). In terms of quality inspection on agricultural products, the parameter setup is an important question for image quality. As low prices of agricultural products have a diverse variety of shapes, sizes, and compositions, a widely accepted parameter setup and protocol are needed to provide automatically by CT scanner.

## 6 CONCLUSIONS

From this literature review, it is proved that X‐ray CT technology is increasingly being employed as an effective tool to identify and describe the quality inspection on agricultural products. More specifically, an extensive range of agricultural products (e.g., wheat, corn, rice, apple, beef) and its applications (e.g., internal quality evaluation, microstructure observation mechanical property measurement, and others) could be investigated with a CT scanner. The popular agricultural crop on CT scan is wheat. The moisture content is the most promising quality feature that can be acquired by X‐ray CT system. The inspection of pesticide residue on agricultural products is difficult for CT scan with current development and possible will be done in.

Besides, the unique advantages of X‐ray CT technology as well as its limitations, compared with other NDT technologies, are also introduced in this paper. A commercial system using portable X‐ray CT with high‐throughput requirements will remain a challenge to accurately inspect in the field. With the improvements in instruments and computational ability, the X‐ray CT technological progress is transformed from external quality to internal quality and single‐item inspection to comprehensive omnidirectional inspection. It is foreseen that the real‐time and high efficient X‐ray CT technology could be used to become indispensable in quality evaluation and product development of agricultural products.

## CONFLICT OF INTEREST

The authors declare that they do not have any conflict of interest.

## ETHICAL APPROVAL

This study does not involve any human or animal testing.

## INFORMED CONSENT

Written informed consent was obtained from all study participants.
